# Do patients care about higher flexion in total knee arthroplasty? A randomized, controlled, double-blinded trial

**DOI:** 10.1186/1471-2474-14-127

**Published:** 2013-04-08

**Authors:** Morten G Thomsen, Henrik Husted, Kristian S Otte, Gitte Holm, Anders Troelsen

**Affiliations:** 1 Department of Orthopedics, University Hospital of Hvidovre, Hvidovre, Denmark

**Keywords:** Knee, Arthroplasty, TKA, Pain, Satisfaction, ROM

## Abstract

**Background:**

Little information exists to support that patients care about flexion beyond what is needed to perform activities of daily living (ADL) after Total knee arthroplasty (TKA). The purpose of this study was to investigate if the achievement of a higher degree of knee flexion after TKA would result in a better patient perceived outcome.

**Methods:**

The study is a randomized, double-blinded, controlled trial in which 36 patients (mean age: 67.2 yrs) undergoing one-stage bilateral TKA randomly received a standard cruciate-retaining (CR) TKA in one knee and a high-flex posterior-stabilized (PS) TKA in the contra lateral knee. At follow-up ROM, satisfaction, pain, “feel” of the knee and the abilities in daily activities were assessed.

**Results:**

At 1-year follow-up we found an expected significantly higher degree of knee flexion of 7° in the high-flex knees (p = 0.001). The high-flex TKA’s showed a mean active flexion of 121°. In both TKA’s the median VAS pain score was 0, the median VAS satisfaction score was 9, and the median VAS score of the patient “feel” of the knee was 9 at 1-year follow-up. Further, there were no significant differences between the knees in the performance of daily activities.

**Conclusions:**

As expected the high-flex TKA showed increased knee flexion, but no significant differences in the patient perceived outcomes were found. This suggests little relevance to the patients of the difference in knee flexion – when flexion is of this magnitude – as pain free ROM and high patient satisfaction were achieved with both TKA’s.

**Trial registration:**

ClinicalTrials.gov: NCT00294528

## Background

When joint arthroplasty was introduced as a treatment for arthritic disease, relief of pain was the primary goal. Since the late 1970’s more attention has been drawn towards restoring normal function. Restoration of knee flexion is an important factor in determining the functional outcome after total knee arthroplasty (TKA). Because of this, range of motion (ROM) is widely used as an outcome measurement to describe the success of treatment. Many efforts have been made to improve ROM including new prosthetic designs (e.g. high-flex prosthetic designs) and postoperative rehabilitation protocols. Studies have shown that high degrees of knee flexion can be achieved following TKA surgery [1-9].

In recent years, however, emphasis has been on patient-derived outcomes such as the ability to complete daily activities and the satisfaction with the outcome of treatment. Previous studies have shown that a minimum of 110 degrees of flexion is needed to complete activities of daily living such as walking normally, rising from a chair and ascending/descending stairs [10,11]. Studies also show that increased flexion beyond 110 degrees leads to increased functional ability [10,12], and Ritter et al. found that patients with ROM of 128 – 132 degrees achieved the best functional results [4]. However, studies have not been able to show that increased flexion beyond 110 degrees have a significant influence on patient satisfaction.

Theoretically and reported in studies posterior stabilized (PS) TKA designs result in higher degrees of flexion than cruciate retaining (CR) designs [1,9]. In this study we aimed to investigate whether patients care about the expected achievement of higher knee flexion after insertion of a high-flex PS TKA compared to a standard CR TKA, in terms of increased patient satisfaction, reduced pain, better “feel” of the knee and better abilities in activities of daily living.

## Methods

**Figure 1:**
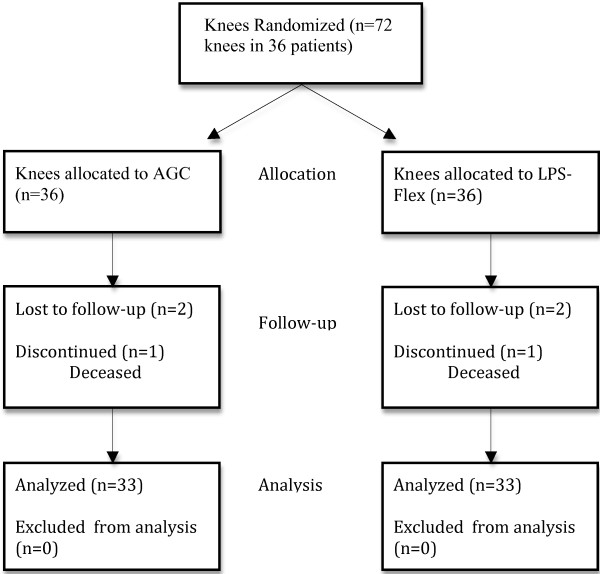
CONSORT flow diagram of 72 knees in 36 patients.

The present study, approved by the local ethics committee (Ethics committee of the Capital Region of Denmark) and registered at Clinicaltrials.gov (NCT00294528), is a prospective, double-blinded randomized study performed in 36 consecutive patients undergoing one-stage bilateral TKA between February 2004 and September 2006. Indications for surgery were bilateral arthritis with disabling pain from both knees. Patients were included in the study following informed consent to bilateral one-stage operation and acceptance of two different TKA’s. Patients were excluded from having a one-stage bilateral procedure if they had a history of or objective findings indicating cardiopulmonary disease (i.e. ASA-score ≥ 3). A total of 36 patients were included in the study. Of these 1 died of unrelated causes before 1-year follow-up and 2 were lost to 1-year follow-up. Thus, the study group consisted of 33 patients (19 females) with a mean age at the time of surgery of 67.2 years (range: 49–84) and a mean BMI of 29.4 (range: 19–41). In 35 patients the diagnosis was osteoarthritis and for 1 patient rheumatoid arthritis. A CONSORT flow diagram for the current investigation is found in Figure 1.

**Figure 2:**
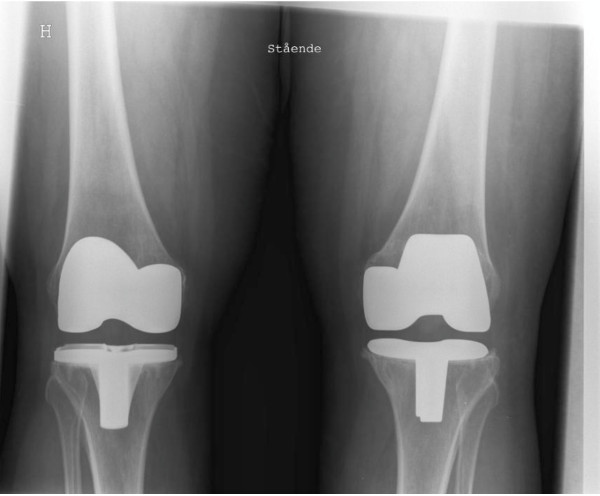
**Anteroposterior radiograph showing the knees of a standing patient 1 year postoperatively. **H: Right knee, LPS-Flex (Zimmer^®^). Left knee, AGC (Biomet^®^).

Each of the 36 patients received a cemented CR AGC TKA (Biomet-Merck, Warsaw, Indiana, USA) in one knee and a cemented PS LPS-Flex TKA (Zimmer, Warsaw, Indiana, USA) in the other knee (Figure 2). These two prosthesis designs were chosen because both have produced excellent clinical results in earlier studies [2,3,6-8,13,14], but given the biomechanical properties of the high-flex PS TKA design, when compared to a standard CR TKA design, we hypothesized that higher degrees of flexion would be achieved in knees receiving the high-flex PS prosthesis [6,7]. The left knee was operated on first and a randomized computer-generated list decided the prosthesis to be used in either knee. Patients were not aware of which knee received which prosthesis.

A senior surgeon performed all surgical procedures. The routine operative technique involved a standard medial para-patellar approach in a bloodless field obtained by the use of a femoral tourniquet (100 mmHg above systolic blood pressure) from incision until cementation was finished. No lateral releases were performed, and patella was resurfaced in all cases. Thorough cleaning of osteophytes from the back of the knee and release of the posterior capsule from the femur was completed in all cases. At the end of surgery all knees had a ROM from full extension until the calf met the thigh. Tranexamic acid [15] was administered routinely. Drains were not used; postoperative cooling was used at the patients’ discretion during hospitalization. All patients received a standardized combined spinal-epidural anesthesia. Oral pain treatment consisted of Paracetamol (1 g x 4), NSAID and opioid at regular intervals and upon request.

Patients left the postoperative recovery ward after a few hours and attempted to mobilize upon arrival at the ward. Physiotherapy was started on the first postoperative day and took place once or twice daily until discharge and followed by a maximum of 8 group sessions at an outpatient physiotherapy clinic. DVT prophylaxis consisted of low molecular weight heparin (LMWH, enoxaparin 40 mg sc) starting 6–8 hours postoperatively and continuing once daily in the evening until discharge. No extended prophylaxis was given and no mechanical devices were used (including compression stockings).

All patients were admitted to a dedicated fast track hip and knee arthroplasty unit. Strictly functional discharge criteria were applied (independency in personal care, ability to walk > 70 m with crutches or better, ability to get in and out of bed and into and up from a chair, sufficient oral pain treatment (VAS < 5 on activity) and acceptance of discharge) and all patients were discharged directly to their homes. Median length of hospital stay was 5 days (range: 4–12).

Preoperative knee extension and flexion was assessed for each knee. Follow-up was performed at 6 weeks, 3 months, 6 months and 1 year postoperatively. All assessments were performed by one investigator blinded to which knee had which prosthesis. The postoperative evaluation consisted of a clinical examination with measurement of active and passive flexion and extension. Measurements were performed with the use of a goniometer. Patients were asked about their ability to ride a bike and squat, and they were asked about barriers in daily living and their ability to kneel for each knee. Pain, satisfaction with the TKA and “feel” of the knee was assessed for each TKA using a visual analog scale (VAS) questionnaire. Satisfaction was defined as how well the knee performed according to patient expectations. “Feel” of the knee was defined as how close to a normal knee the TKA felt, e.g. stability and smoothness in movement. At 1-year follow-up, patients filled in the Short Form-36 (SF-36) questionnaire which is a validated questionnaire consisting of 36 questions reviewing the patients’ physical and mental health related QoL [16]. One patient was revised before final follow-up due to deep infection in the knee that received the standard CR prosthesis. Data from this patient are included in the study until revision.

Data that are normally distributed are presented as mean values and ranges, and comparisons are made using a two-sample t-test. Data that are not normally distributed are presented as median values and ranges, and comparisons are made using the two-sample Wilcoxon rank-sum (Mann–Whitney) test. Binomial data are presented as proportions or percentages, and comparisons made by Pearson’s chi-square test. The level of significance was set at p < 0.05. For all statistical analyses the STATA statistical package, version 10.1 (College Station, Tx, USA) was used.

An a priori sample size calculation revealed that a significant difference (p < 0.05) of 1 point in patient satisfaction (SD: ± 1.5) could be shown with 80% power if 36 knees of each type (36 patients) were included.

## Results

### Range of motion

Preoperatively the mean maximum flexion did not differ significantly between the two groups: 116° for the CR TKA’s and 118° for the high-flex PS TKA’s. At 6 weeks and 3 months follow up, no statistical differences in flexion, both active and passive, were found between the two TKA’s. At 6 months and 1-year follow-up, however, the high-flex PS TKA’s showed significantly increased flexion compared to the CR TKA’s both actively and passively (Table 1). At 1-year follow-up, 30 of the CR TKA’s and 31 of the high-flex PS TKA’s were able to extend to 0–5 degrees, no significant difference.

**Table 1:** Results for flexion (active and passive), knee pain, satisfaction with knee and feel of knee preoperatively and at 3 months, 6 months and 1 year follow up

**Type of prosthesis**	**Pre-operative**	**3 months**	**6 months**	**1 year**
	**Flexion – passive (mean (range))**			
Standard CR TKA	116 (range 70–140)	115 (range 90–133)	117 (range 105–130)	120 (range 104–146)
High-flex PS TKA	118 (range 80–140)	119 (range 70–135)	123 (range 105–138)	127 (range 107–146)
p-value	0.61	0.18	0.0012	0.0012
	**Flexion – active (mean (range))**			
Standard CR TKA	-	109 (range 90–125)	111 (range 96–128)	114 (range 90–135)
High-flex PS TKA	-	113 (range 70–130)	115 (range 98–130)	121 (range 105–140)
p-value	-	0.14	0.04	0.0011
	**Knee pain (median (range))**			
Standard CR TKA	-	2 (range 0–5)	2 (range 0–5)	0 (range 0–8)
High-flex PS TKA	-	2 (range 0–9)	1 (range 0–7)	0 (range 0–8)
p-value	-	0.43	0.85	0.94
	**Satisfaction with knee (median (range))**			
Standard CR TKA	-	8 (range 4–10)	8 (range 4–10)	9 (range 3–10)
High-flex PS TKA	-	8 (range 1–10)	9 (range 3–10)	9 (range 4–10)
p-value	-	0.50	0.56	0.61
	**Feel of the knee (median (range))**			
Standard CR TKA	-	8 (range 2–10)	8 (range 2–10)	9 (range 0–10)
High-flex PS TKA	-	7 (range 0–10)	8 (range 0–10)	9 (range 0–10)
p-value	-	0.30	0.64	0.53

### Knee pain

The postoperative pain scores, according to the VAS-score, did not differ between the two TKA’s at any time of follow-up (Table 1).

### Satisfaction with the TKA

There were no differences in patient satisfaction with the TKA between the CR TKA’s and the high-flex PS TKA’s at any time of follow up (Table 1).

### “Feel” of the knee

No significant differences in feel were recorded at any time of follow-up (Table 1).

### Ability to perform activities of daily living

At 1-year follow-up, 31 of 33 patients were able to ride a bike and 20 of 33 patients were able to squat. 22 patients reported no barriers in daily living according to the CR TKA, compared to 23 for the high-flex PS TKA (p = 0.79). 31 patients were able to kneel with the CR TKA, and 30 patients were able to kneel with the high-flex PS TKA (p = 0.64). The number of patients that were satisfied with the achieved ROM during activities of daily living was 29 and 32 for the CR and PS TKA’s, respectively (p = 0.16).

### Short form (SF)-36

26 of 33 patients completed the SF-36 questionnaire at 1-year follow-up. The median physical component score (PCS) was 47.8 (range: 20.6-57.9). The median mental component score (MCS) was 59.2 (range: 32.8-64.6).

## Discussion

Many efforts have been made to increase ROM after TKA, but despite the clinical success of TKA in achieving high ROM, little information can be found in the literature regarding the potential association between objective parameters such as ROM and subjective parameters such as patient satisfaction, and feel of the knee. The present study was performed to determine if an association could be found between objective measurements of increased ROM and subjective parameters of ability to complete activities of daily living, pain, feel of the knee and satisfaction with the knee when high degrees of flexion (beyond 110 degrees) are achieved after TKA.

One of the strengths of this study is the use of patients operated on bilaterally. The use of two treatments in the same patient has the advantage that patient-related factors (i.e. BMI, thigh-calf index, pain threshold) are eliminated, thereby improving the quality of analysis. Is it fair to use two different prostheses? In a study on two different prostheses in bilaterally operated patients, the PS and CR TKA were equally preferred among patients – indicating similar outcomes of feel and satisfaction [5]. Also, it was stated that this method could be the only way to detect the potential subtle differences between prostheses – hence we included the not validated “feel” of the prosthesis.

Did we achieve high flexion? In our study we found that the use of a high-flex PS prosthesis resulted in a significantly increased flexion ability of 7 degrees, both active and passive, when compared to a standard CR TKA at 1-year follow-up. Our results support the data presented by Bin et al. and Weeden et al. [2,3] who found that higher knee flexion can be achieved when using a high-flex design. In the present study we compare a high-flex PS prosthesis to a standard CR prosthesis. To our knowledge no previous study has compared these designs in achieving high flexion and although it may be argued that a PS-design may give better flexion in weight bearing, the primary goal in this study was not to investigate if the use of a high-flex prosthesis would result in an increased postoperative ROM when compared to a standard prosthesis, but to test if a higher postoperative knee ROM would result in an increased patient satisfaction and better overall patient perceived outcomes.

To our knowledge only a few previously published studies have investigated the relationship between physical findings such as ROM and patient-derived factors such as ADL, satisfaction and “feel” of the knee.

Devers et al. [10] conducted a retrospective study in which 122 TKA’s were divided into 3 groups depending on their passive flexion 1 year post-operatively. Low flexion was defined as flexion < 110 degrees, medium flexion was defined as 110–130 degrees and high flexion as flexion > 130 degrees. When comparing the medium and high flexion group, they found a positive correlation between the degree of postoperative flexion and fulfillment of expectations, “feel” of knee and functional ability. In contrast, patient satisfaction was not influenced by the degree of postoperative flexion in this study. Two other studies performed by Padua et al. and Ritter et al. [17,18] also found a positive correlation between increased postoperative flexion, both active and passive, and ability to perform activities of daily living. However, no significant correlation between flexion and patient satisfaction and pain was found.

Unlike Devers et al., Padua et al. and Ritter et al. [13,17,18] we found no association between the increased postoperative flexion found in the high-flex group and postoperative knee pain, satisfaction with the TKA, “feel” of the knee and the ability to perform activities of daily living. In our study, the postoperative flexion was equal to or exceeded 95 degrees in all TKA’s, and therefore our results are supported by the data published by Miner et al. [19] who in a prospective randomized study found no relationship between ROM and ability to perform ADL when flexion exceeded 95 degrees.

The results for SF-36 physical and mental component scores found postoperatively in this study are comparable to results obtained in similar studies of health-related quality of life after TKA [20-24], and to normative SF-36 scores found in the Danish population the age of 65–74 years: PCS 48,39 (mean), MCS 58,54 (mean) [16]. Thus, combined outcomes of both knee designs produce outcomes comparable to other designs and to non-operated age-matched persons – again indicating no limitations in either prosthesis for gaining quality of life.

Some methodological limitations to this study should be acknowledged: First, we did not include a joint specific knee score. Instead, we used the SF-36 questionnaire to assess the health related quality of life one year postoperatively. Second, the follow-up period of 12 months was relatively short and we can draw no conclusions about long-term wear and satisfaction. Earlier studies, however, have revealed that ROM and patient satisfaction reaches a plateau beyond 1 year [6,25,26], and therefore we believe that our results can be used as a good marker for long-term function and satisfaction.

## Conclusions

In conclusion we performed a randomized, double-blinded, controlled clinical trial in which 33 patients, who underwent bilateral TKA with a high-flex PS prosthesis in one knee and a standard CR prosthesis in the other knee, were available for follow up at 12 months. We found that the use of the high-flex PS prosthesis resulted in a significantly increased flexion of 7 degrees compared to the standard CR prosthesis. The increased flexion, however, did not reflect on patient-derived parameters such as pain, satisfaction with the result and feel of knee. This suggests little relevance to patients of the difference in knee flexion – when flexion is of this magnitude – as pain free ROM, good knee function and high patient satisfaction were achieved with both TKA’s.

## Competing interests

None of the authors have any financial and personal relationships with other people or organizations that could inappropriately influence their work.

## Authors’ contributions

MGT summarized all data and drafted the manuscript. HH conceived the study, participated in its design and coordination, carried out surgical procedures and helped to draft the manuscript. KSO carried out surgical procedures. GH Collected postoperative data and AT performed the statistical analysis and helped to draft the manuscript. All authors have read and approved the final manuscript.

## Pre-publication history

The pre-publication history for this paper can be accessed here:

http://www.biomedcentral.com/1471-2474/14/127/prepub
